# Optimal Center of Rotation for Ball-And-Socket Thumb Carpometacarpal Arthroplasty Identified Using Three-Dimensional Kinematic Analysis: A Pilot Study

**DOI:** 10.3389/fbioe.2022.868150

**Published:** 2022-06-01

**Authors:** Atsuro Murai, Akihiro Kurosawa, Kaoru Tada, Hiroshi Tachiya, Atsuya Tamai, Mika Akahane, Masashi Matsuta, Yuta Nakamura, Hiroki Kawashima, Hiroyuki Tsuchiya

**Affiliations:** ^1^ Department of Orthopaedic Surgery, Graduate School of Medical Science, Kanazawa University, Kanazawa, Japan; ^2^ Institute of Science and Engineering, Faculty of Mechanical Engineering, Kanazawa University, Kanazawa, Japan; ^3^ Faculty of Health Sciences, Institute of Medical, Pharmaceutical and Health Sciences, Kanazawa University, Kanazawa, Japan

**Keywords:** arthroplasty, thumb, carpometacarpal, osteoarthritis, ball-and-socket implant, kinematics, center of rotation

## Abstract

Total joint arthroplasty is one of the surgical option for thumb carpometacarpal (CMC) joint arthritis, however the optimal position the center of rotation (COR) has not been quantified. The purpose of this study is to identify ideal ball-and-socket thumb carpometacarpal joint implants and the optimal position of the COR. We obtained eight right thumb computed tomography images each from ten healthy men, comprising four images each of thumbs at various angles of flexion-extension and abduction-adduction. We reconstructed 3D bone models on 3D CAD, created virtual ball-and-socket implants with three variables (neck offset, implant height, neck rotation), and found the optimal COR where the position change in the COR was smallest across various thumb positions. When the offset was 4.5 mm, neck rotation angle was 130.6° from the radial side to the palmar side of the first metacarpal, and implant height from the distal end of the metacarpal was 43.6 mm, we could restore almost normal kinematics. This study could serve as a reference for implant development and surgical technique guidelines.

## Introduction

Thumb carpometacarpal (CMC) joint osteoarthritis is the second most common type of hand osteoarthritis. Surgical treatment is recommended if there is no improvement after conservative treatment (Facca and Liverneaux, 2012). Although there are various surgical methods, a Cochrane review comparing seven surgical methods revealed that no surgical procedure was particularly good (Wajon et al., 2015).

Total joint arthroplasty is a surgical option for thumb CMC osteoarthritis (OA). Several review articles have reported better early improvements in pain and patient-reported outcome measures relative to those with trapeziectomy, including ligament reconstruction, but complications were more common with a prosthesis (Verhulst et al., 2020; Holme et al., 2021). Although a wide variety of prostheses have been made for a long time, there are currently no recommended single implants (Huang et al., 2015; D'Agostino et al., 2018). Among available implants, the ball-and-socket prosthesis is now mainly used (D'Agostino et al., 2018) and achieves good results, with a survival rate of approximately 90% at 10 years reported recently (Dumartinet-Gibaud et al., 2020). However, the most important complications are implant loosening and dislocations (Facca and Liverneaux, 2012; Ganhewa et al., 2019; Holme et al., 2021), and it may be difficult to continue using CMC prostheses in the United Kingdom unless the surgical results improve (Huang et al., 2015).

Although there are various reasons for dislocation and loosening, such as fracture of the trapezium, metallosis, mechanical impingement between the prosthetic neck and the cup, or imperfect cup placement (Facca and Liverneaux, 2012; Vitale et al., 2013; Brauns et al., 2019), it is important to place prostheses in the correct position (Duerinckx and Caekebeke, 2016; Cootjans et al., 2017) and restore normal CMC joint kinematics (Lerebours et al., 2020) using a prosthesis in order to prevent complications, such as loosening or dislocation of the prosthesis (Lerebours et al., 2020). However, the surgical methods for placement of prostheses are based on expert experience (Duerinckx and Caekebeke, 2016) and the optimal position for the implant has not yet been determined (Brauns et al., 2019). In addition, it is often said that replacing the CMC joint with a single center of rotation (COR) is very difficult without changing the normal thumb CMC kinematics because the original saddle joint has a variable COR (de Raedt et al., 2013; Huang et al., 2015; D'Agostino et al., 2018).

We assumed that the reason why ball-and-socket CMC implants cannot restore normal kinematics is that the optimal COR and placement position have not been determined. The aim of this study was to investigate the optimal COR for ball-and-socket CMC joint implants when replacing the saddle joint with a ball joint by analysing the kinematics of a healthy thumb CMC joint using three-dimensional (3D) computer-aided design (CAD). In addition, we investigated the morphology of the ideal ball-and-socket CMC joint implant that can be placed at the optimal COR, and evaluated the reproducibility of normal CMC kinematics after total joint arthroplasty using the ideal implant.

## Materials and Methods

### Defining the Optimal Center of Rotation and Ideal Ball-And-Socket Implant

We defined the optimal COR as the position where the position change in the COR was smallest for various thumb positions. We also defined an ideal implant as an implant that can be placed at the optimal COR.

### Study Subjects and Computed Tomography Scanning

**FIGURE 1:**
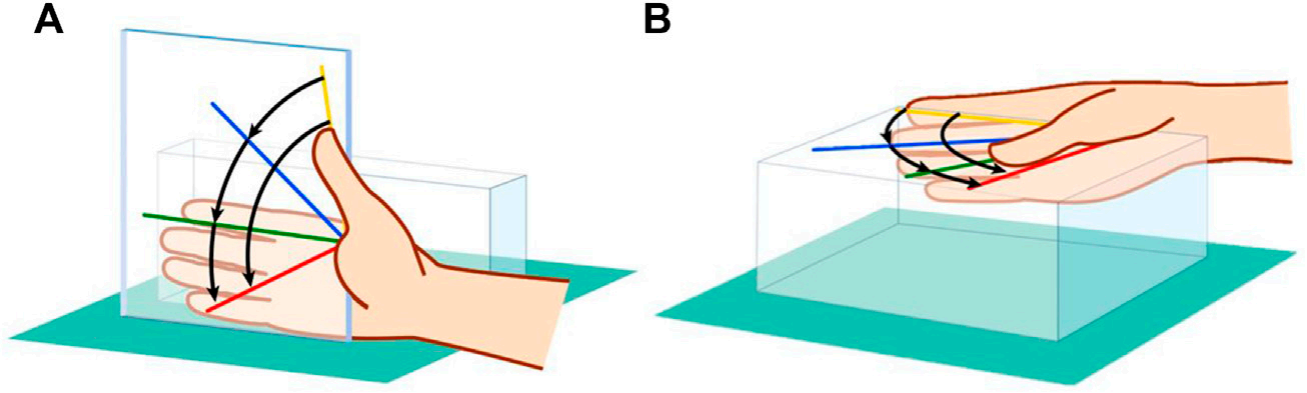
Eight-position CT imaging method using a polycarbonate rig. **(A)** Maximum extension to maximum flexion positions. **(B)** Maximum adduction to maximum abduction positions.

The study complied with the 1975 Helsinki Declaration Ethics Guidelines and was approved by the institutional review board of the authors’ affiliated institutions. The subjects were ten healthy men (23–32 years old; average, 26.8 years old) who provided informed consent. Using a polycarbonate rig as previously reported (D'Agostino et al., 2017; Tada et al., 2021), we obtained eight static right thumb computed tomography (CT) images statically: four of the thumb in positions ranging from maximum extension position to maximum flexion position (positions 1–4), and four ranging from maximum adduction to maximum abduction (positions 5–8) (Figure 1). A 128-slice multidetector CT was used in this study. The scan range was from the right wrist joint to the tip of the thumb in each of the eight thumbs. The CT acquisition parameters were as follows: tube voltage, 100 kV; tube current, 20 mAs; CT dose index, 0.82 mGy; pitch factor, 0.5; and slice thickness, 0.75 mm. The radiation dose was estimated to be 0.004 mSv per scan. No arthritic changes on CT were observed in any of the subjects.

### 3D Bone Model Reconstruction

First, bone was extracted from the CT images following binarization using 3D-Slicer (ver 4.4.0), a free open-source software platform for biomedical research. Unnecessary pixel areas were manually deleted. A 3D model of each bone in the STL file format was obtained from 3D Slicer. Second, using 3D CAD software, the 3D model in the STL file format was made into a solid model, and a 3D model of the thumb CM joint that could be analysed was created.

### Definitions of Coordinate System

We defined the first metacarpal and trapezium bones coordinate axis in the same way as in previous reports (Halilaj et al., 2013; Crisco et al., 2015a).

**FIGURE 2:**
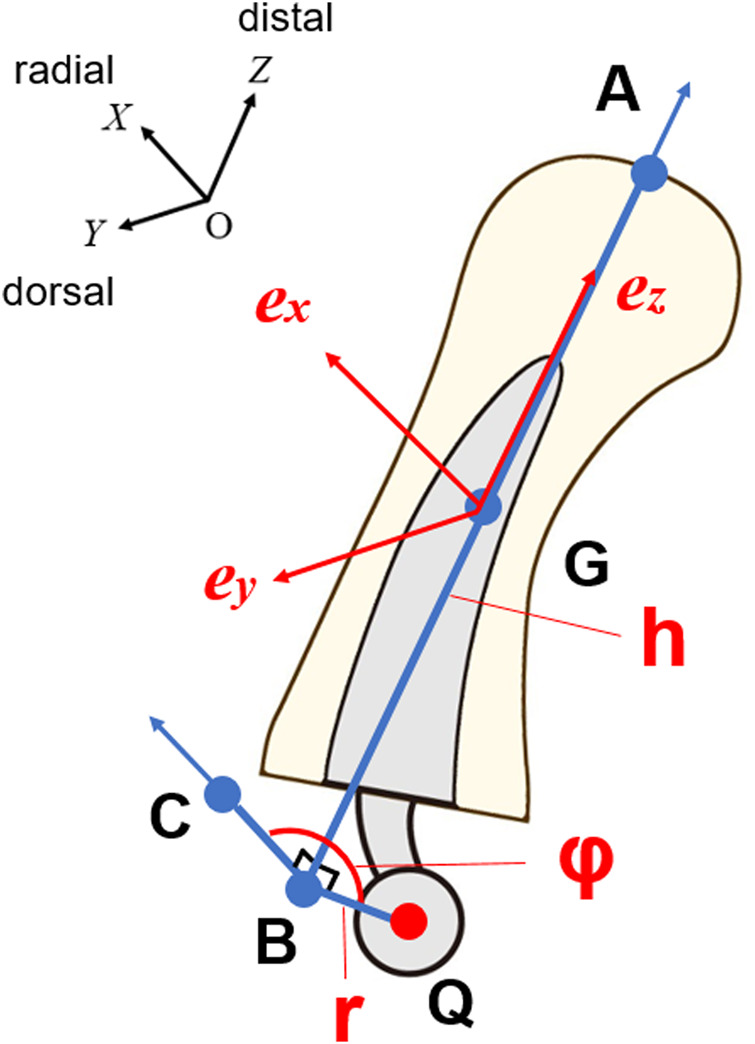
Coordinate system for virtual ideal ball-and-socket implants. AB: bone axis, G: center of gravity, C: point at distance r from point B in positive *X* axis direction, e: unit vectors from center of gravity to coordinate axis.

We represented the metacarpal in the O-*XYZ* coordinate system and the trapezium in the $O^{\prime }-X^{\prime }Y^{\prime }Z^{\prime }$ coordinate system. Origin O was set as the center of gravity of the metacarpal and origin $O^{\prime }$ as the center of gravity of the trapezium. Although the positive *X* direction is volar, the positive *Y* direction is proximal, and the positive *Z* direction is radial in the previous reports; to perform mathematical analysis, we defined the positive axis of *X* as radial, the positive axis of *Y* as dorsal, and the positive axis of *Z* as distal (Figure 2). The *Z* axis is the metacarpal axial COR, and we defined the *Z* axis as the metacarpal bone axis.

### Design of the Virtual Ideal Ball-And-Socket Implant

To analyse the COR, we created a virtual ball-and-socket implant that can reconstruct the optimal COR with three variables. The variables were defined as *r* (the offset distance from the implant axis to the COR), *h* (the height from the distal end of the metacarpal to the center of the head), and *φ* (the neck rotation angle from the radial side of the metacarpal). For angle *φ*, the palmar side was positive (Figure 2). We defined the implant axis as the axis of the metacarpal.

The COR for each position was designated as point Q. In addition, we defined the distal end of the metacarpal bone on the *Z* axis as point A, the point at the height of the implant on the *Z* axis as point B, and the point at a distance *r* from the point B in the positive *X* axis direction as point C. Using the three variables, distance *r* = BQ = BC, AB = *h*, and angle CBQ = *φ* and points B and C can be expressed as in Equations 1, 2 (Figure 2).

$$\mathrm{OB}=\mathrm{OA}+h\left(-e_{z}\right)$$ (1)

$$\mathrm{OC}=\mathrm{OB}+re_{x}$$ (2)

In addition, the center of gravity of the metacarpal bone was defined as point G, and the unit vectors from the center of gravity to the coordinate axis in the positive direction of the metacarpal bone were defined as ***e***_***x***_, ***e***_***y***_, and ***e***_***z***_.

Using these variables, the coordinates of the COR *Q* can be obtained using Rodrigues’ rotation formula as in Equations 3, 4, where ***R*** denotes a rotation matrix, C*φ* means cos*φ*, S*φ* means sin*φ*, and V*φ* means 1-cos*φ*.

$$Q\left(x,y,z\right)=R\left(e_{z},-\varphi \right)\mathrm{OC}=R\left(e_{z},-\varphi \right)\left\{re_{x}+h\left(-e_{z}\right)+\mathrm{OA}\right\}$$ (3)

$$R\left(e_{z},-\varphi \right)=\left(\begin{array}{ccc} e_{zx}^{2}V_{-\varphi }+C_{-\varphi } & e_{zx}e_{zy}V_{-\varphi }-e_{yy}S_{-\varphi } & e_{zx}e_{zz}V_{-\varphi }+e_{zy}S_{-\varphi }\\ e_{zx}e_{zy}V_{-\varphi }+e_{zz}S_{-\varphi } & e_{zy}^{2}V_{-\varphi }+C_{-\varphi } & e_{zy}e_{zz}V_{-\varphi }-e_{zx}S_{-\varphi }\\ e_{zx}e_{zz}V_{-\varphi }-e_{zy}S_{-\varphi } & e_{zy}e_{zz}V_{-\varphi }+e_{zx}S_{-\varphi } & e_{zz}^{2}V_{-\varphi }+C_{-\varphi } \end{array}\right)$$ (4)

Thus, *Q* can be represented by the coordinates OA determined at each metacarpal position and *r*, *h*, *φ*.

### Analysis of Optimal Center of Rotation

First, we added a virtual ideal ball-and-socket implant to each of the eight 3D metacarpal bone models. Second, we set *Q*_*i*_ (*X*_*i*_, *Y*_*i*_, *Z*_*i*_) (*i* = 1–8) as the coordinates of the COR for each thumb position. If all *Q*_*i*_ positions are in the same coordinate, it means complete reconstruction of the native thumb CMC movement with the artificial joint. Third, to calculate the optimal COR position, we calculated the average COR coordinate $\bar{Q}$ (Eq. 5). Fourth, we calculated the distance between the COR *Q*_*i*_ and the average COR $\bar{Q}$ , and calculated the variance (*V*) of the distance between *Q*_*i*_ and $\bar{Q}$ . The variance, *V*, is the variation in the COR depending on the thumb motion, so the change in position of the COR is smallest when *V* is lowest. *V* can be expressed as shown in Eq. 6. We calculated *r*, *h*, and *φ* when *V* was lowest and determined the ideal virtual ball-and-socket implant.

$$\bar{Q}=\left(\bar{x},\bar{y},\bar{z}\right)=\left(\frac{1}{n}\sum_{n=1}^{n}x_{i},\frac{1}{n}\sum_{n=1}^{n}y_{i},\frac{1}{n}\sum_{n=1}^{n}z_{i}\right)\;\left(i=1∼8\right)$$ (5)

$$V=\frac{1}{n}{\sum_{n=1}^{n=8}\left(Q_{i}-\bar{Q}\right)}^{2}=\frac{1}{n}\sum_{n=1}^{n=8}\left[{\left(x_{i}-\bar{x}\right)}^{2}+{\left(y_{i}-\bar{y}\right)}^{2}+{\left(z_{i}-\bar{z}\right)}^{2}\right]$$ (6)

### Evaluation of Normal Motion Reconstruction With Ideal Ball-And-Socket Implants

We created new metacarpal 3D bone models with ideal virtual ball-and-socket implants using a fixed optimal COR on 3D CAD. Each new metacarpal model was created using a method in which the distal end of the metacarpal bone was located at the bone axis of the original metacarpal.

By overlaying 3D bone models of the metacarpal bone before and after arthroplasty, the volume matching portion (%) and the difference in the centroid (mm) of the metacarpal and the angle change (degree) of the metacarpal bone axis before and after arthroplasty were measured to evaluate the difference between the true *in vivo* position versus the position constrained to a single COR. The volume matching portion was determined by the ratio of the matching volumes of the two 3D bone models from the original metacarpal volumes.

## Results

**FIGURE 3:**
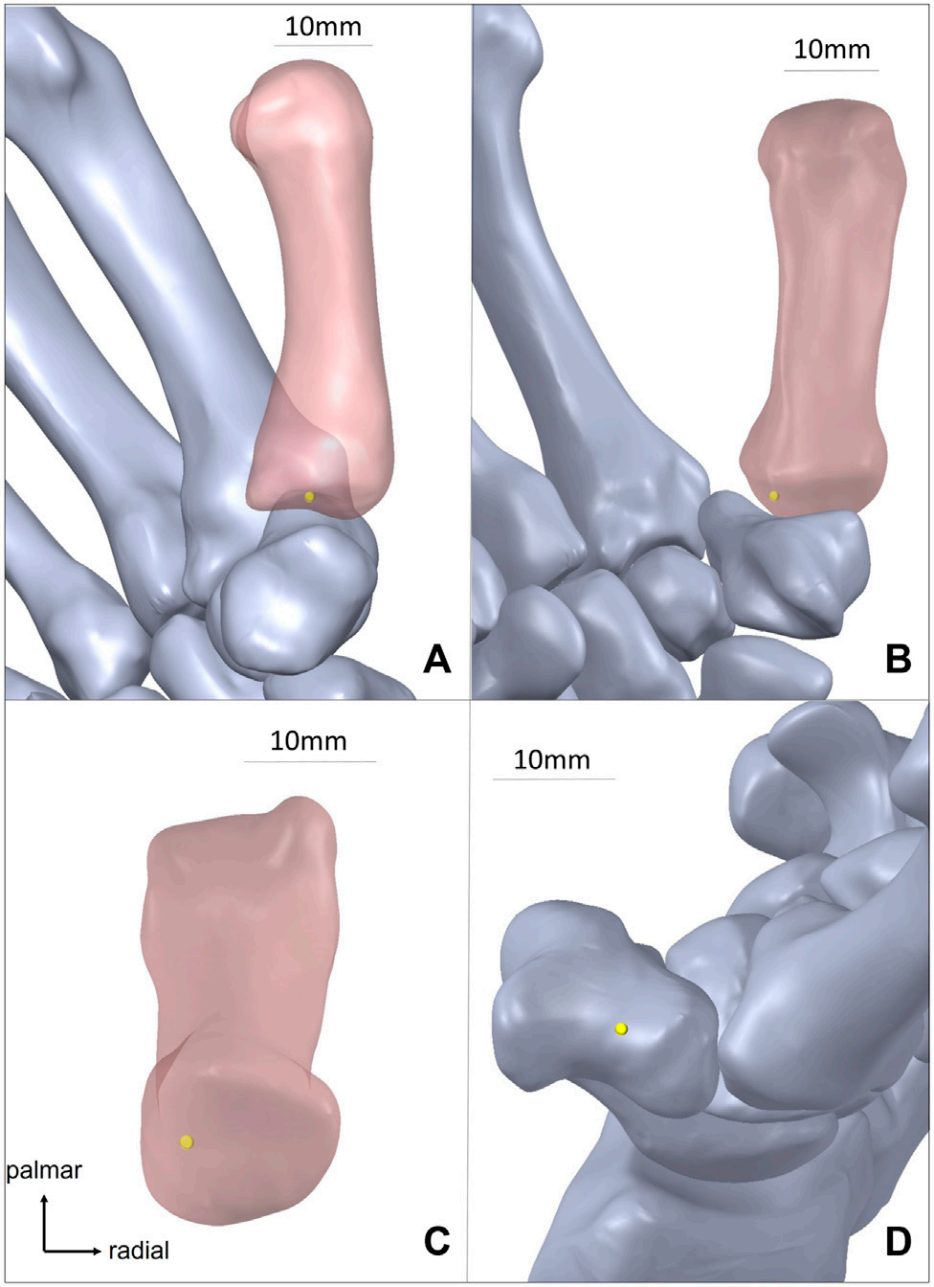
Optimal center of rotation in 3D bone models (yellow point is the optimal center of rotation). **(A)** Optimal center of rotation of 1st metacarpal after arthroplasty from radial side. **(B)**. Optimal center of rotation of 1st metacarpal after arthroplasty from palmar side. **(C)** Model of 1st metacarpal bone from proximal side. **(D)**. Optimal center of rotation without 1st metacarpal.

When *r* was 4.5 ± 1.69 (mean ± standard deviation) mm, *φ* was 130.6 ± 12.4°, and *h* was 43.6 ± 2.37 mm, the change in position of the eight centers of rotation after arthroplasty were the smallest. The mean metacarpal length in 10 CT scans was 46.0 ± 1.24 mm, and *h* was 2.4 mm shorter than the metacarpal bone length. In all 10 cases, each h was less than each metacarpal length, and COR was located in the metacarpal. In the trapezium coordinate system, the COR was located distal to the trapezium distal joint surface and slightly on the palmar and ulnar sides of the trapezium (Figure 3). The standard deviation of the COR coordinate position variation in 10 cases was 1.00 mm in the *X* axis direction, 1.76 mm in the *Y* axis direction, and 1.42 mm in the *Z* axis direction.

**FIGURE 4:**
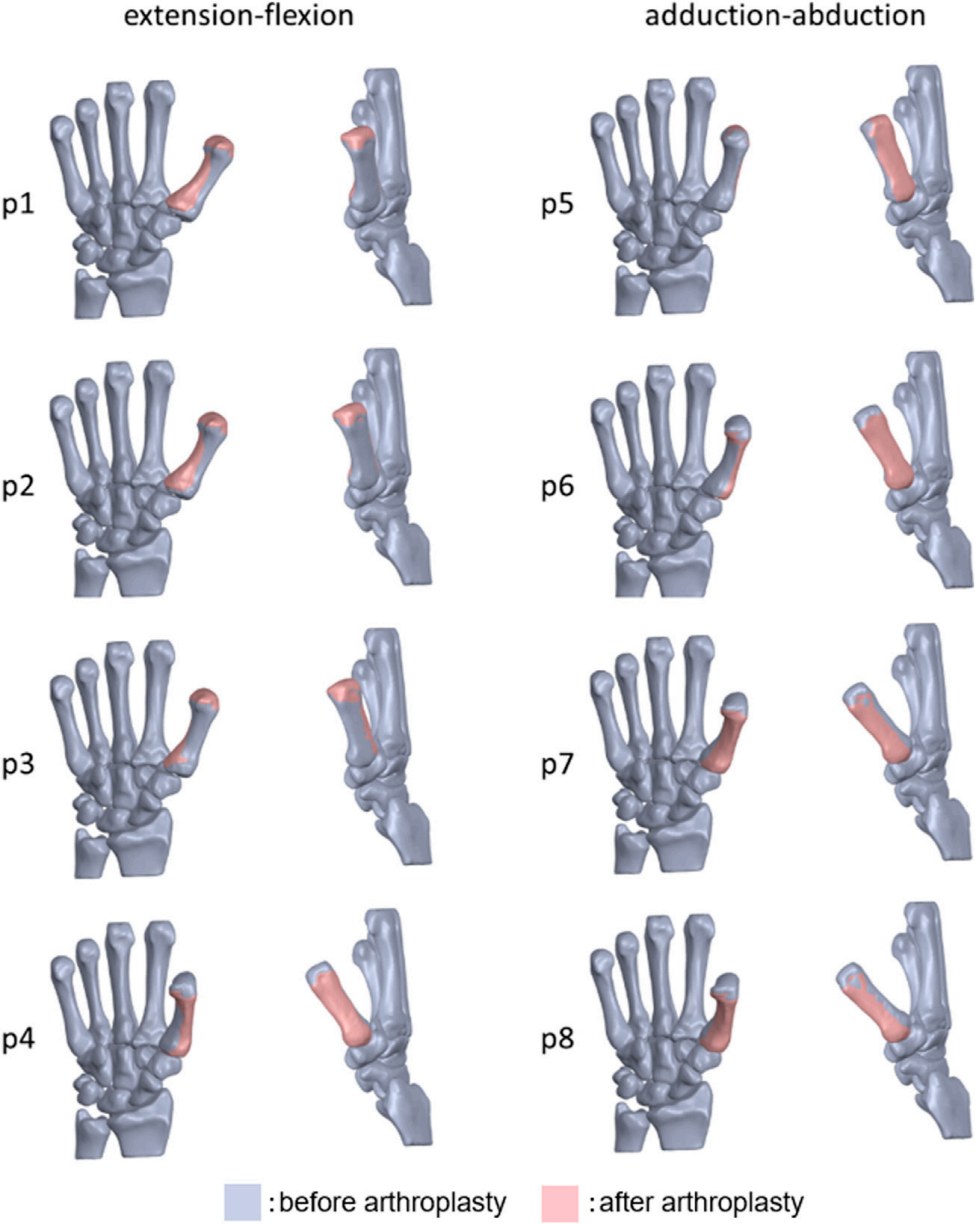
Overlapped 3D bone models of the 1st metacarpal bone before and after arthroplasty of one subject (p1: maximum extension, p4: maximum flexion, p5: maximum adduction, p8: maximum abduction).

**FIGURE 5:**
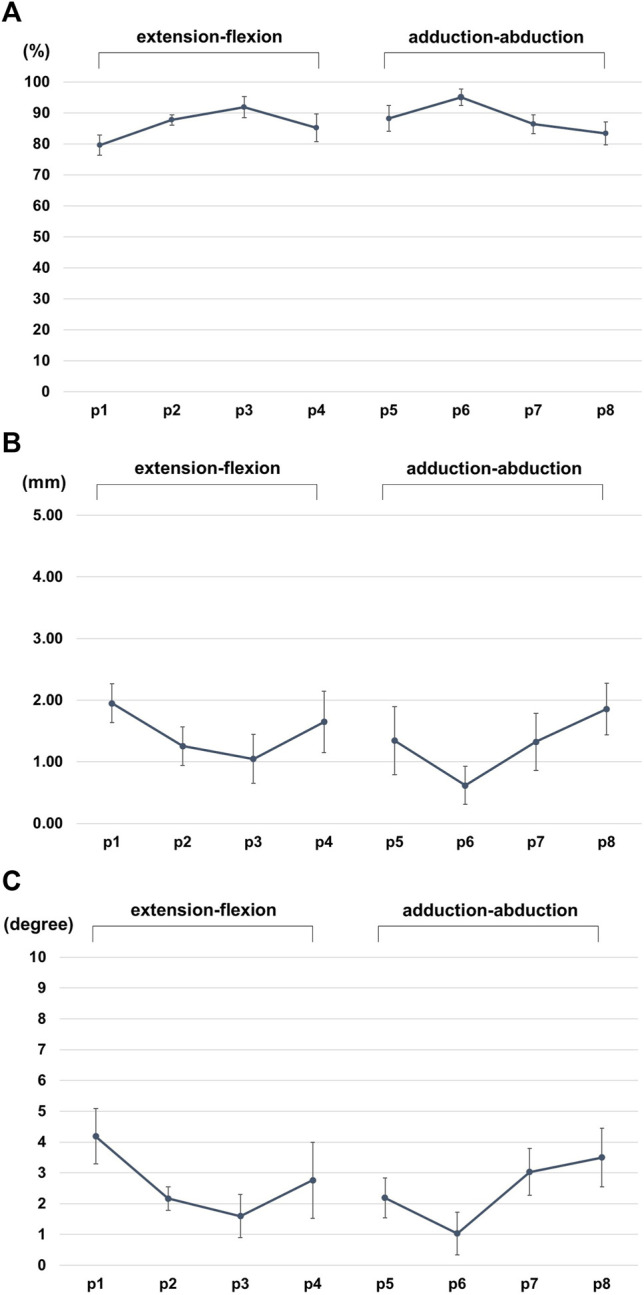
**(A)** Volume matching portion (%) of the metacarpal before and after arthroplasty. **(B)** the difference in the centroid (mm) of the metacarpal. **(C)** Angle change (degrees) of the metacarpal bone axis before and after arthroplasty.

The volume matching portion of the metacarpal before and after arthroplasty was 86.1 ± 5.55% in the extension-flexion motions, 88.3 ± 5.47% in the adduction-abduction motions; the mean volume matching portion of all positions was 87.2 ± 5.61% (Figure 4, Figure 5A).

The difference in the centroid of the metacarpal bone before and after arthroplasty in each position changed by 1.50 ± 0.52 mm for extension-flexion motions and 1.30 ± 0.63 mm for adduction-abduction motions, and the mean difference in the two centroid for all positions was 1.40 ± 0.58° (Figure 5B).

The angle change in the metacarpal bone before and after arthroplasty in each position changed by 2.68 ± 1.29° for extension-flexion motions and 2.43 ± 1.22° for adduction-abduction motions, and the mean angle change for all positions was 2.56 ± 1.26° (Figure 5C).

## Discussion

In our study, ideal implants that restore native thumb CMC kinematics had an offset distance of 4.5 mm, the neck rotation was 130.6° from the radial side to the palmar side, and the COR was located 2.4 mm more distal than the proximal end of the metacarpal bone. By placing the ideal ball-and-socket implant at the optimal COR, 87.2% of the volume of the normal metacarpal bone can be reproduced, the difference in the centroid of the metacarpal was only 1.4 mm, and the change in the bone axis before and after arthroplasty was only 2.56°.

The purpose of this study is to determine the optimal COR. However, Crisco et al. reported that the extension-flexion rotation axis located in the trapezium, the abduction-adduction rotation axis located in the first metacarpal, and translational movements also occurred during these movements, indicating that the motion of thumb CMC joint is not determined by a single COR. Huang et al. reported that ball and socket implants couldn’t restore normal CMC joint kinematics. If we were to pursue the restoration of normal kinematics further, the use of a saddle joint prosthesis aimed at anatomical reconstruction (Uchiyama et al., 1999) would be a consideration; however, they are currently not used because of their poor results (Lerebours et al., 2020). Therefore, we investigated the COR that reproduce the most normal kinematics possible with the best-performing balls and sockets at present. Our hypothesis was that if a single COR was used, the optimal COR would be the average of each thumb position.

In a previous study on optimal placement of the ball-and-socket prosthesis, Ledoux et al. reported that the cup should be placed in the center of the trapezium to prevent trapezium fracture, and there are many reports on placement using that approach (Ledoux, 1997). Duerinckx et al. studied implant placement using fluoroscopy and found that the cup should be placed parallel to the proximal articular surface of the trapezium (Duerinckx and Caekebeke, 2016). Caekebeke et al. performed thumb CMC arthroplasty by placing a cup parallel to the proximal articular surface of the trapezium and reported a survival rate of 96% at a mean of 65 months (Caekebeke and Duerinckx, 2018). Blauns et al. studied which cup angle prevents dislocation using ARPE ball-and-socket implants and fresh cadavers (Brauns et al., 2019). They confirmed that it is important to place a cup parallel to the proximal articular surface of the trapezium, as Duerinckx mentioned. However, these studies targeted ball-and-socket implants in which the COR is located at the trapezium, and there has not been a study on the restoration of normal kinematics with correct placement.

In our study, which did not limit the COR to the trapezium, the optimal COR was located in the metacarpal bone and not in the trapezium. It is difficult to place prostheses at the optimal COR using the most commonly used implants in which the COR is located at the trapezium. Cooney et al. have previously reported a cement-type reverse ball-and-socket thumb CMC joint, the “Mayo” implant, where the COR is located in the metacarpal (Cooney et al., 1987). Lerebours et al. reported that the reverse COR, located in the metacarpal, has not been shown to cause complications (Lerebours et al., 2020). Total hip arthroplasty, which uses similarly ball-and-socket prosthesis, has been reported to reduce dislocation and wear by replacing the COR at the anatomical hip center (Watts et al., 2016) (Kim et al., 2017). For these reason, the use of the reverse type thumb CMC prosthesis might be a new option for further improvement of the thumb CMC joint arthroplasty.

In this study, we pursued the ideal optimal COR for 3D CAD without considering ligaments and bony impingement. Nevertheless, it was difficult to restore 100% normal kinematics with the optimal COR that we investigated. However, even for total hip arthroplasty, where good clinical results have been obtained with ball-and-socket implants, the postoperative femoral position has not been completely restored (Tsai et al., 2014). We believe that the results of ball-and-socket implants for thumb CMC osteoarthritis cannot be completely denied, even if they are not 100% reproducible.

This study had some limitations. First, we did not consider soft tissues, such as ligaments or impingement of the implant. Therefore, further studies are needed to determine whether dislocation can be prevented. Second, in patients who undergo surgery, the tension in the tendon changes due to degeneration of the joint; therefore, further study is needed to determine whether dislocation can be prevented. Since our goal in this study was to restore normal kinematics, we used healthy hands in our study. Third, although thumb CMC joint arthritis is common in women, we only targeted men because of the radiation exposure required during CT imaging. According to previous research on bone morphology and kinematics of the thumb CMC joint of men and women, although there is a difference in bone size between men and women, the morphology and kinematics are not significantly different (Crisco et al., 2015a). We believe that the results of this study can be applied to women by adjusting for differences in size. Fourth, we restricted the thumb motion to extension-flexion and adduction-abduction; therefore, the presence of normal motion in other positions, such as the opposition, was unclear. It is reported that various limb positions of the thumb can be reproduced by combining adduction-abduction and extension-flexion movements (Crisco et al., 2015b; D'Agostino et al., 2017). We hope that the results of this study will be useful in various thumb movements, such as opposition, but we believe that further studies are needed.

This study is the first to investigate the optimal positioning of ball-and-socket thumb CMC joint arthroplasty using 3D CT bone models, and we report new indicators for the COR, implant offset, and neck rotation, which have not had a clear standard until now.

As a result of this study, we found that the optimal COR is placed in the metacarpal; however, we do not think that current implants are non-functional, as the COR is located in the trapezium. In future, we would like to study the optimal COR for common ball-and-socket implants where the COR is located in the trapezium. This study is the first to investigate the optimal position of ball-and-socket thumb CMC joint arthroplasty using 3D CT bone models, and we report new indicators for the COR, implant offset, and neck rotation, which have not had clear standards to date. Although further research is needed, we hope that this study will contribute to improve the performance of thumb CMC joint arthroplasty.

The error bars represent the standard deviation.

## Informed Consent

“Informed consent was obtained from all individual participants included in the study.”

## Data Availability

The raw data supporting the conclusions of this article will be made available by the authors, without undue reservation.
